# Dose-ranging and further therapeutic evaluation of a bicistronic humanized TrkB-BDNF gene therapy for glaucoma in rodents

**DOI:** 10.1186/s44477-025-00003-y

**Published:** 2025-08-18

**Authors:** Andrew Osborne, Tasneem Z. Khatib, Michael Whitehead, Terrance Mensah, Sadat Yazdouni, Bart Nieuwenhuis, Zara Ali, Jared Ching, Robert Watt, Naoki Kishi, Naoki Kozono, James R. Tribble, Peter S. Widdowson, Keith R. Martin

**Affiliations:** 1 John Van Geest Centre for Brain Repair, Department of Clinical Neurosciences, University of Cambridge, Cambridge, UK; 2 Quethera Ltd, Babraham Research Campus, Cambridge, UK; 3 Ikarovec Ltd, The Norwich Research Park Innovation Centre, Norwich, UK; 4 Eye Department, Addenbrooke’s Hospital, Cambridge, UK; 5 Medical Sciences Division, University of Oxford, Oxford, UK; 6 Ophthalmology, Byers Eye Institute at Stanford, Stanford University, Stanford, CA USA; 7 Cambridge Institute for Medical Research, University of Cambridge, Cambridge, UK; 8 Astellas Pharma Inc. Miyukigaoka, Tsukuba, Ibaraki Japan; 9 School of Optometry and Vision Sciences, Cardiff University, Cardiff, UK; 10 Department of Clinical Neuroscience, Division of Eye and Vision, St. Erik Eye Hospital, Karolinska Institutet, Stockholm, Sweden; 11 Centre for Eye Research Australia, and Royal Victorian Eye and Ear Hospital, Melbourne, Australia; 12 Ophthalmology, Department of Surgery, University of Melbourne, Melbourne, Australia

**Keywords:** BDNF, TrkB, Glaucoma, Neuroprotection, Gene therapy

## Abstract

**Background:**

Glaucoma is a leading cause of irreversible blindness, characterized by the progressive degeneration of retinal ganglion cells (RGCs). Activation of the Tropomyosin receptor kinase B (TrkB) pathway by mature brain-derived neurotrophic factor (mBDNF) has emerged as a promising neuroprotective strategy, given its critical role in promoting RGC survival in preclinical models.

**Methods:**

We advanced the development of a bicistronic adeno-associated viral (AAV) gene therapy vector engineered to co-express human TrkB and mBDNF. The vector was optimized with fully human transgene sequences and evaluated for functional expression and dose scalability to support clinical translation. Expression tracking and efficiency were enhanced by incorporating a self-cleaving 2A peptide sequence.

**Results:**

In a mouse model of optic nerve crush, intravitreal administration of 1.52E8 to 7.60E8 genome copies (GC)/eye significantly mitigated RGC damage. In a rat model of laser-induced ocular hypertension, doses ranging from 3.80E8 to 1.90E9 GC/eye preserved both visual function and RGC survival. The 2A peptide facilitated efficient co-expression of TrkB and mBDNF while minimizing interference from endogenous protein pathways.

**Conclusions:**

These findings demonstrate that co-expression of human TrkB and mBDNF via a bicistronic AAV vector yields robust, dose-dependent neuroprotection and sustained transgene expression in two distinct models of glaucomatous injury. This gene therapy represents a promising first-in-class candidate for the treatment of glaucoma.

**Supplementary Information:**

The online version contains supplementary material available at 10.1186/s44477-025-00003-y.

## Background

Glaucoma is a leading cause of irreversible blindness worldwide, primarily characterized by the progressive degeneration of retinal ganglion cells (RGCs), resulting in a gradual loss of vision. Lowering intraocular pressure (IOP) with drugs, laser treatment or surgery is the mainstay of current glaucoma treatment, but many individuals with glaucoma continue to progress to blindness despite access to IOP lowering therapy [1]. The absence of effective therapies either to prevent RGC loss that continues despite effective IOP reduction, or to reverse RGC loss, remains a critical concern for both clinicians and patients [2]. This underscores the urgent need for innovative treatment strategies that directly target diseased or dysfunctional neurons.

Gene therapy has emerged as a powerful approach to treat various genetic disorders, with gene-agnostic strategies being increasingly investigated for prevalent blinding conditions such as age-related macular degeneration, geographic atrophy, and diabetic macular edema [3, 4]. These therapies offer the potential for sustained therapeutic effects and reduced treatment burden, addressing the limitations of current management options. However, gene therapies for glaucoma have yet to progress to clinical trials, with several translational challenges identified in bringing neuroprotective strategies for glaucoma from the laboratory to clinical practice [5].

Among the emerging neuroprotective strategies for glaucoma [6–9], the activation of the Tropomyosin receptor kinase B (TrkB) receptor represents a promising approach. Experimental models consistently demonstrate that TrkB receptor agonists, such as mature brain-derived neurotrophic factor (mBDNF), can mitigate RGC loss, preserve visual function, and enhance RGC survival [10–12]. The development of novel TrkB agonists holds potential for facilitating long-term survival signaling and improving treatment outcomes [13–15], and we have previously demonstrated that a combination of TrkB/BDNF therapy is more effective than administering either mBDNF or TrkB alone [16, 17].

In this study, we extend previous work on TrkB and mBDNF co-expression as a bicistronic gene therapy for retinal neuroprotection [16–18]. Building on this foundational research, we developed and re-evaluated a humanized version of the construct, TrkB-2A-mBDNF, designed for progression into investigational new drug (IND)-enabling studies and future clinical translation for glaucoma. We demonstrate that the humanized construct retains robust expression and activation, with dose-dependent neuroprotective efficacy in two established rodent models of RGC injury, helping to define scalable minimum effective and maximum tolerated doses for larger animal studies.

These advances seek to address the critical unmet need for effective gene therapies in glaucoma, with the ultimate goal of improving patient outcomes and mitigating the progression of this blinding disease.

## Materials and methods

### Plasmids

Murine and human TrkB-2A-mBDNF plasmids were cloned using standard molecular biology techniques at GenScript (Piscataway, USA) based on the previously described construct [18] and patent US11471539B2. A plasmid map of the new human TrkB-2A-mBDNF plasmid is shown in Supplementary Fig. 1A with codon optimization of DNA sequences performed using the on-line tool www.idtdna.com/CodonOpt to achieve an average GC content of 56.88%.

### HEK293T cell culture

HEK293T cells (12,022,001, Sigma-Aldrich), were cultured in 6-well plates or on 13 mm glass coverslips coated with 10 µg/mL poly-L-lysine (P4707, Sigma-Aldrich). Cells were maintained in Dulbecco’s minimum essential medium (DMEM) supplemented with 10% fetal bovine serum (FBS), 1% penicillin, and 1% streptomycin until they reached 80% confluence. The medium was then replaced with DMEM without additives, and cells were transfected with 4 µg plasmid DNA and 4 µL/mL Lipofectamine 2000 (12,566,014, Thermo Fisher Scientific) for 24 h at 37 °C. TrkB antagonist ANA-12 (5,063,040,001, Sigma-Aldrich) was administered at concentrations of 10 µM or 100 µM post-transfection for 1 h.

### Measurement of secreted BDNF

Secreted BDNF was quantified in the culture medium 25 h post-transfection. The medium was centrifuged to remove debris and analyzed using a commercial Human BDNF ELISA kit (RAB0026, Sigma-Aldrich). The concentration of BDNF was determined by comparison to freshly prepared BDNF standards.

### Animals

Adult C57BL/6 J mice (30 g, Charles River) and adult Sprague Dawley rats (150-200 g, Charles River) were housed in groups of five per cage. Following a 7-day acclimatization period at the University of Cambridge Phenomics Animal Facility, animals were randomized and assigned to various procedure groups.

### Test compound

Human TrkB-2A-mBDNF was manufactured as an AAV2 vector at Astellas Pharmaceutical Science & Technology Labs using proprietary technology. Controls were either platform buffer or Null vector (7.60E8 GC/eye, Vigene Biosciences USA), depending on the study. AAV2 Human TrkB-2A-mBDNF was titred via qPCR against the inverted terminal repeats (ITRs) and used at the following doses after a single freeze–thaw cycle:

#### Mice

- Mid dose: 1.52E8 Genome Copies (GC)/eye
- High dose: 7.60E8 GC/eye

#### Rats

- Low dose: 9.50E7 GC/eye
- Mid dose: 3.80E8 GC/eye
- High dose: 1.90E9 GC/eye

Rat vector doses were allometrically scaled from those selected for mice, based on vitreal volume (mice: 5.3 µL, rats: 13.4 µL), total retinal area (mice: 15.6 mm^2^, rats: 52.5 mm^2^), and axial ocular diameter (mice: 3.7 mm, rats: 6.3 mm), resulting in an overall scaling of ~ 2.5-fold.

### Intravitreal delivery of AAV

Mice and rats were anesthetized with an intraperitoneal injection of ketamine (100 mg/kg) and xylazine (10 mg/kg), with topical 1% tetracaine (Bausch & Lomb) used as a local anesthetic. Using an operating microscope, 2 µL (mice) or 5 µL (rats) of AAV vector was bilaterally injected through the sclera into the vitreous of each eye, approximately 3 mm posterior to the superior-temporal limbus (Syringe: 5 µL, #65RN; Needle: ga33, 8 mm, pst2, Hamilton). Injections were administered over 20 seconds to allow diffusion of the vector suspension and intraocular pressure equilibration before needle removal. The cornea was punctured prior to needle removal to minimize reflux [19], and ocular lubricant (Carbomer, Medicom Healthcare Ltd) was applied immediately post-procedure. Vectors were allowed to express for at least 21 days before further investigation.

### Electroretinography (ERG)

Full-field ERGs were recorded simultaneously from both eyes of mice or rats under injectable anesthesia, as previously described [16, 17]. Animals were dark-adapted overnight (> 12 h), and ERG recordings were conducted under low-level red-light illumination. Pupil dilation was achieved using 0.5% tropicamide (Bausch & Lomb) and 2.5% phenylephrine (Bausch & Lomb). Light stimuli were applied using an Espion E3 system with a full-field Ganzfeld sphere (Diagnosys). Scotopic threshold responses were recorded at −4.73 log cd.s.m^–2^, with peak amplitude between 80–120 ms taken as the positive scotopic threshold response (pSTR) amplitude. The B-wave was recorded at −1.90 log cd.s.m^–2^, and A-wave recordings were measured at 1.29 log cd.s.m^–2^.

### Optic Nerve Crush (ONC)

Mice were subjected to unilateral ONC as previously detailed [17, 20, 21]. Seven days post-ONC, mice were perfused transcardially with 4% paraformaldehyde (PFA) (1040031000, Sigma-Aldrich) under terminal anesthesia, and eyes were collected for retinal flatmount processing.

### Laser-Induced IOP Elevation

Ocular hypertension was unilaterally induced in rats using a modification of the method developed by Levkovitch-Verbin et al. (2002) [22], which has been extensively used in-house. Intraocular pressure (IOP) was elevated through forty to sixty laser pulses (wavelength: 532 nm, spot size: 50 µm, power: 700 mW, duration: 600 ms) directed around the circumference of the trabecular meshwork to impair aqueous drainage. Two laser procedures, one week apart, resulted in a longer duration of elevated IOP suitable to induce RGC functional and morphological damage [17, 23, 24]. Contralateral eyes served as injected internal controls. IOP was measured bilaterally under anesthesia on procedure days and while awake at subsequent time points using a TonoLab rebound tonometer (Tiolat Oy).

### Human retinal tissue preparation

Retinal tissue samples were obtained from formalin-fixed, postmortem human eyes donated to the School of Optometry and Vision Sciences, Cardiff University from the Minnesota Lions Eye Bank. A total of 20 eyes were utilized, comprising 10 from patients diagnosed with glaucoma and 10 from age-matched control individuals (Supplementary Data 2A).

For each eye, four retinal samples were dissected with precision. Two samples were taken from the central retina, in proximity to the macula, while the other two were collected from regions with documented visual field loss (exampled in Supplementary Fig. 2B). These regions were identified based on visual field tests conducted during the patients'last clinical appointment prior to death. Control eye samples were dissected from corresponding locations to those of the glaucomatous eyes, to allow for direct comparison between diseased and healthy tissue.

All samples were handled under standardized conditions to minimize tissue degradation and stored appropriately until further processing.

### Ocular Section Immunohistochemistry (IHC)

#### Sample Collection and Preparation

Rodent eyes were collected following CO2 inhalation and post-fixed in 4% PFA at 4 °C overnight whilst human retinas had already been stored in formalin long term. The eyes were then dehydrated in 30% sucrose (S0389, Sigma-Aldrich) in PBS at 4 °C for over 24 h. Subsequently, the eyes were embedded in silicone molds containing optimal cutting temperature (OCT) compound (4583, Sakura Finetek). The eye globes were frozen on dry ice and sectioned at 13 μm thickness through the dorsal–ventral/superior-inferior axis of the retina using a cryostat (OTF 5000, Bright Instruments). Sections were mounted onto Superfrost™ Plus slides (10149870, Fisher Scientific). HEK293T cells grown on coverslips for comparison were washed twice in PBS and fixed for 30 min in 4% PFA in PBS at room temperature.

### Immunostaining

Ocular sections (and HEK293T cells) were simultaneously blocked and permeabilized by incubation in 5% normal goat serum (NGS) (G9023, Sigma-Aldrich) in PBS with 0.3% Triton X-100 (10421871, Fisher Scientific) and 2% bovine serum albumin (BSA) (A7906, Sigma-Aldrich) for 60 min at room temperature. Sections were then incubated overnight at 4 °C with primary antibodies (Table 1) diluted in blocking solution, followed by incubation with secondary antibodies (Table 1) diluted in blocking solution for 120 min at room temperature. Nuclei were counterstained with DAPI (62248, Fisher Scientific) and mounted with FluorSave™ Reagent (345789-20 M, Millipore).

**Table 1:** Antibodies

*Antibody host and target*	*Catalogue no., supplier*	*Technique*	*Dilution*
Rabbit anti-TrkB	ab33655, Abcam	Immunofluorescence Western blot	1:500 1:500
Rabbit anti-TrkB	4603, Cell Signaling Technologies (CST)	Western blot	1:500
Rabbit anti-p-TrkB (Y515)	PA536695, Thermo Fisher	Immunofluorescence Western blot	1:300 1:500
Rabbit anti-BDNF	ab108319, Abcam	Immunofluorescence Western blot	1:500 1:500
Mouse anti-2A	NBP259627, Novus Biologics	Immunofluorescence Western blot	1:500 1:500
Rabbit anti-p-ERK (Thr202/Tyr204)	4370, CST	Immunofluorescence Western blot	1:500 1:500
Rabbit anti-t-ERK	4695, CST	Western blot	1:500
Rabbit anti-p-Akt (Ser473)	9271, CST	Immunofluorescence Western blot	1:500 1:500
Rabbit anti-t-Akt	4691, CST	Western blot	1:500
Guinea pig anti-RBPMS	1832, PhosphoSolutions	Immunofluorescence	1:500
Mouse anti-TUJ1	G7121, Promega	Immunofluorescence	1:400
Chicken anti-MAP2	ab5392, Abcam	Immunofluorescence	1:1000
Rabbit anti-GFAP	Z0334, DAKO	Immunofluorescence	1:500
Guinea Pig anti-IBA1	234004, Synaptic Systems	Immunofluorescence	1:500
Rabbit anti-β-actin	4967, CST	Western blot	1:1000
Goat anti-mouse IgG (H + L) Secondary Antibody, Alexa Fluor 647	A21235, Invitrogen	Immunofluorescence	1:1000
Goat anti-mouse IgG (H + L) Secondary Antibody, Alexa Fluor 555	A21424, Invitrogen	Immunofluorescence	1:1000
Goat anti-guinea pig IgG (H + L) Secondary Antibody, Alexa Fluor 555	A21435, Invitrogen	Immunofluorescence	1:1000
Goat anti-rabbit IgG (H + L) Secondary Antibody, Alexa Fluor 488	A11034, Invitrogen	Immunofluorescence	1:1000
Goat anti-guinea pig IgG (H + L) Secondary Antibody, Alexa Fluor 647	ab150187, Abcam	Immunofluorescence	1:500
Goat anti-chicken IgG (H + L) Secondary Antibody, Alexa Fluor 568	A11041, Invitrogen	Immunofluorescence	1:1000
HRP goat anti-rabbit IgG secondary antibody (Peroxidase)	PI-1000, Vector Laboratories	Western blot	1:8000
HRP goat anti-mouse IgG secondary antibody (Peroxidase)	PI-2000, Vector Laboratories	Western blot	1:8000

### Imaging

Sections and cells were imaged using an epifluorescence microscope (DM6000, Leica Microsystems). High magnification images were obtained using confocal microscopy with a 40X oil objective, 1.5X digital zoom, and a 1.0–1.4 μm sequential scanning z-step interval (SP5, Leica Microsystems).

### Ocular Section IHC Expression Profiling

#### Quantification

Quantification of TrkB, 2A, BDNF, microtubule-associated protein 2 (MAP2), and glial fibrillary acidic protein (GFAP) was performed at eight positions per retina from four images. Images were oriented so that the retinal layers ran vertically, with the RGC layer on the left-hand side. A fixed region of interest spanning a retinal depth of 205 µm by a width of 35 µm was applied through individual RGCs, and plot profile measurements were taken for the required channels. Data points were collated, with eight plot profiles representing a single retina.

### Area Under the Curve (AUC) Measurements

AUC measurements were recorded from the mean plot profiles focusing on the RGC layer (0-40 µm depth) for TrkB, 2A, and BDNF, and the inner plexiform layer (40-80 µm depth) for MAP2. The trapezoidal rule was used to calculate the AUC, with the x-axis representing depth through the retinal layers and the y-axis representing immunofluorescence intensity.

### GFAP and IBA1 Quantification

GFAP immunoreactivity was assessed in six positions spanning a larger area (205 µm x 100 µm) of the retina. Manual counting was performed to quantify ionized calcium-binding adaptor molecule 1 (IBA1) positive cells within the RGC/nerve fiber layer (NFL), as well as the inner plexiform layer (IPL) and inner nuclear layer (INL).

### Human retina measurements

DAPI-labeled RGC layer counts and IPL quantification were performed using six images per retinal sample, captured across a 600 µm distance to confirm pathology (Supplementary Fig. 2C). For quantification of TrkB and p-TrkB expression in RGCL cells, individual RGCs were traced in the images to delineate their boundaries. Fluorescent intensity values corresponding to TrkB and p-TrkB were obtained and aggregated across 200–300 RGCs per data point for each sample, ensuring a comprehensive representation of protein expression across the retina.

### Retinal Flatmount Processing

#### Preparation and dissection

Eyes designated for retinal flatmounts were processed three to six hours after perfusion fixation. Dissection was performed in PBS, with the cornea, ciliary body, and lens removed using Dumont #5 Forceps (11295–10, Fine Surgical Tools). The retina was gently separated from the underlying retinal pigment epithelium, and any residual vitreous was removed from the RGC layer using a fine paintbrush (#B074Z6X76D, Amazon). Flatmounts were post-fixed in 4% PFA for 1 h and stored in 500 µL PBS in individual wells of a 24-well plate, kept in the dark at 4 °C until all retinas were collected.

### Immunostaining

Mouse flatmounts were washed in PBS (3 × 15 min) and simultaneously blocked and permeabilized by incubation in 3% NGS in PBS with 0.3% Triton X-100 and 1% BSA for 60 min at room temperature. Rat retinal flatmounts were dissected into two equal halves, with one half used for RGC quantification and the other half processed for sectioning.

Flatmounts were incubated overnight at 4 °C with primary antibodies (Table 1) diluted in blocking solution with gentle rocking. After washing in PBS (3 × 15 min), retinas were incubated with secondary antibodies (Table 1) diluted in blocking solution for 180 min at room temperature with gentle rocking. Nuclei were counterstained with DAPI.

### Imaging and quantification

RBPMS-labelled RGCs were imaged using a 20X objective from four central and four peripheral regions of each mouse retina, as previously described [17]. For rat half retinas, 12 images were taken. RGC counts were measured by a blinded third-party using ImageJ and the Image-based Tool for Counting Nuclei (ITCN) plugin (https://imagej.net/ij/plugins/itcn.html). ITCN settings were: width 30 pixels, minimum distance 10 pixels, threshold 0.1. RBPMS counts were expressed as density of RGCs/mm^2^.

Higher resolution, representative images were obtained using a #SP5 confocal microscope with a 40X oil objective, 1.5X digital zoom, and a 1.0–1.4 μm sequential scanning z-step interval. TUJ1 was included as an alternative RGC marker if RBPMS staining was unsuccessful.

### Western blotting

#### Sample preparation

Vector-injected animals were euthanized via CO_2_ inhalation. The eyes were opened, and the retinas were carefully dissected and frozen on dry ice before storage at −20 °C. Retinas were lysed using 120 µL/retina (mouse) or 250 µL/retina (rat) of freshly prepared Lysis-M reagent containing cOmplete™ Mini EDTA-free Protease Inhibitor Cocktail (04693159001, Roche) and Halt™ Phosphatase Inhibitor Single-Use Cocktail (78428, Fisher Scientific). HEK293T cells were lysed in 350 µL buffer/well. Following 20 min of homogenization with pestles, tissues were centrifuged at 13,000 rpm for 10 min to isolate the soluble cell extract.

### Protein quantification and blotting

Protein concentration was determined using the Pierce™ BCA Protein Assay Kit (23225, Fisher Scientific). Equal quantities of protein were loaded into 4–12% Bis–Tris gels (NP0322BOX, Fisher Scientific). Membranes were blocked in 5% dried skimmed milk in PBS with 0.2% Tween20 (P9416, Sigma-Aldrich) for 60 min and incubated overnight at 4 °C with primary antibodies (Table 1).

Primary antibodies were visualized with HRP secondary antibodies (Table 1) and signal detection and imaging performed using the Amersham ECL Prime Western Blotting Detection Reagent (RPN2232, GE Healthcare) and an Alliance Western blot imaging system (UVItec Ltd).

### Stripping and re-probing

Membranes were stripped using a mild stripping buffer (15 g glycine, 1 g SDS, 10 mL Tween20, pH 2.2, 1L H_2_O) for 20 min at room temperature, followed by washing, re-blocking, and re-incubation with new primary antibodies. Band intensities were quantified using the Gel Plot Profiler tool in ImageJ [25]. For signaling pathway analysis, phosphorylated ERK and Akt levels were normalized to their corresponding total protein levels, consistent with standard methodology for evaluating endogenous pathway activation.

Expression levels of TrkB, mBDNF, and 2A were normalized to β-actin, used as a loading control. For p-TrkB, normalization was also performed against β-actin rather than total TrkB. This is due to the fact that total TrkB is exogenously overexpressed from the viral vector, resulting in dynamic and non-physiological variation in receptor levels. In such cases, normalizing p-TrkB to total TrkB can lead to misinterpretation, as both values are simultaneously affected by vector expression. Therefore, p-TrkB levels are presented relative to β-actin to more accurately reflect the net increase in activated TrkB signaling within treated retinas compared to controls.

### Statistics

Data are presented as mean ± SEM. Student’s T-test was used to compare control and Human TrkB-2A-mBDNF data, including uninjured to injured ERG pSTRs (GraphPad Prism 9.5). Remaining data were analyzed using one-way ANOVA followed by Tukey’s or Dunnett’s modified t-tests for multiple comparisons where p ≤ 0.05. Specific statistical analyses are detailed in the figure legends.

## Results

### Humanization of the TrkB-2A-mBDNF construct

The first stage of this study involved the humanization and codon optimization of the Tropomyosin receptor kinase B (TrkB) construct, with the goal of enhancing its clinical applicability. The murine and human TrkB sequences displayed 97.6% similarity and 94.6% identity (Uniprot P15209 vs Q16620), with the majority of amino acid differences located within the extracellular domain of the receptor (Supplementary Fig. 1B). In contrast, the mature brain-derived neurotrophic factor (mBDNF) sequence exhibited 100% homology between rodents and humans, eliminating the need for any modifications (Uniprot P21237 vs P23560) (Supplementary Fig. 1C).

**Fig. 1:**
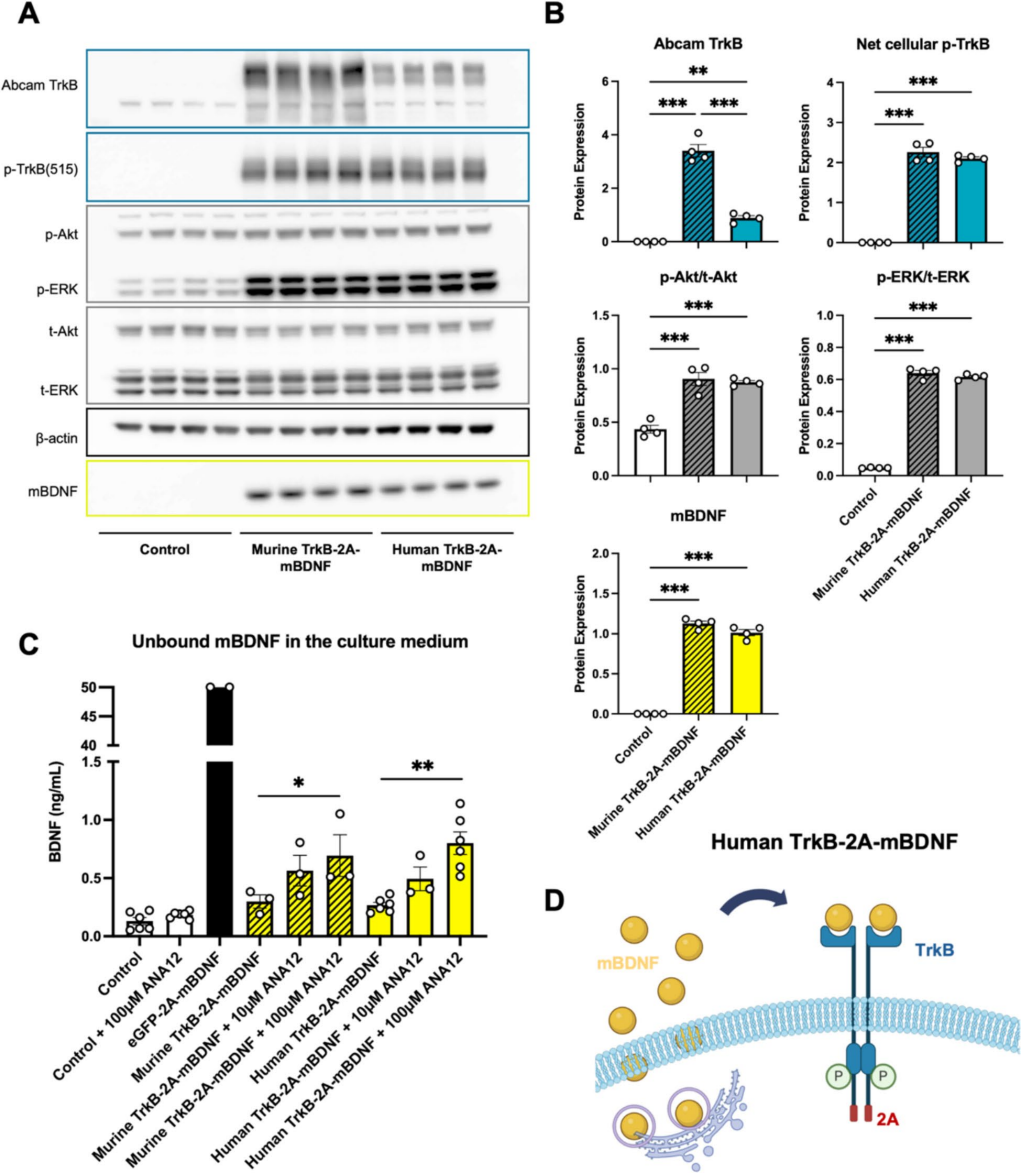
Comparison of murine and human TrkB-2A-mBDNF expression and signaling in HEK293T cells, 24 h post plasmid transfection. A) Western blot analysis of HEK293T cell lysates 24 h post-transfection with TrkB-2A-mBDNF constructs. Blots show the expression of transgenes (TrkB and mBDNF) and activation of downstream signaling pathways, including phosphorylated TrkB (p-TrkB), phosphorylated ERK (p-ERK), and phosphorylated Akt (p-Akt). The control group consists of cells transduced with AAV2-eGFP. B) Quantification of protein expression normalized to β-actin (for TrkB p-TrkB and mBDNF), t-ERK for p-ERK, and t-Akt for p-Akt, with n = 3 for each group. C) Detection of secreted BDNF in the culture medium, measured with or without the presence of TrkB receptor antagonist ANA12. Data points represent n = 2–6 biological replicates, showing the impact of TrkB inhibition on extracellular BDNF levels. D) Schematic representation depicting the mechanism by which secreted mBDNF binds to TrkB receptors on the cell surface, promoting receptor dimerization and intracellular phosphorylation (P) of TrkB. The binding reduces the amount of"free"mBDNF available in the extracellular environment. *p ≤ 0.05, **p ≤ 0.01, ***p ≤ 0.001 (Student’s t-test). Scale bar = 100 µm. Image D created in BioRender

Notably, amino acid substitutions in the TrkB receptor influenced its recognition by the Abcam antibody (ab33655) (Fig. 1A, B), necessitating the evaluation of alternative antibodies targeting different domains of the receptor (Supplementary Fig. 3A). Among the tested antibodies, the Cell Signaling Technology (CST) antibody #4603 demonstrated consistent detection of both murine and human TrkB proteins with comparable accuracy (Supplementary Fig. 3A).

To confirm the functional integrity of the humanized TrkB receptor, downstream signaling was assessed by measuring phosphorylated TrkB (p-TrkB (515)), ERK, and Akt pathways. Both human and murine TrkB-2A-mBDNF constructs significantly elevated downstream signaling activity compared to controls, with no substantial differences between the rodent and human versions (Fig. 1A, B). Specific signaling activity was further visualized in transfected cells (Supplementary Fig. 3B), and quantification of p-Akt and p-ERK protein expression relative to total Akt and ERK revealed that the humanized TrkB construct was as functionally potent as the murine counterpart (Fig. 1B), indicating that detection challenges were due to antibody limitations rather than expression deficiencies.

Importantly, modifications to the TrkB transgene did not affect mBDNF expression between the murine and humanized constructs, as shown by equivalent levels of BDNF protein (Fig. 1A, B). Furthermore, released BDNF was able to bind human TrkB receptors, as demonstrated by reduced levels of unbound mBDNF in the culture medium when TrkB was co-expressed, compared to control transfections with eGFP (Fig. 1C). The application of the non-competitive TrkB antagonist ANA-12 at 100 µM inhibited TrkB binding and BDNF internalization, leading to an increase in unbound BDNF levels in the medium. These findings corroborate earlier construct development data [18], further illustrated by a schematic representation of BDNF binding to TrkB receptors on the same or neighboring cells (Fig. 1D).

## Short-term safety and expression analysis of Human TrkB-2A-mBDNF in the mouse retina

**Fig. 2:**
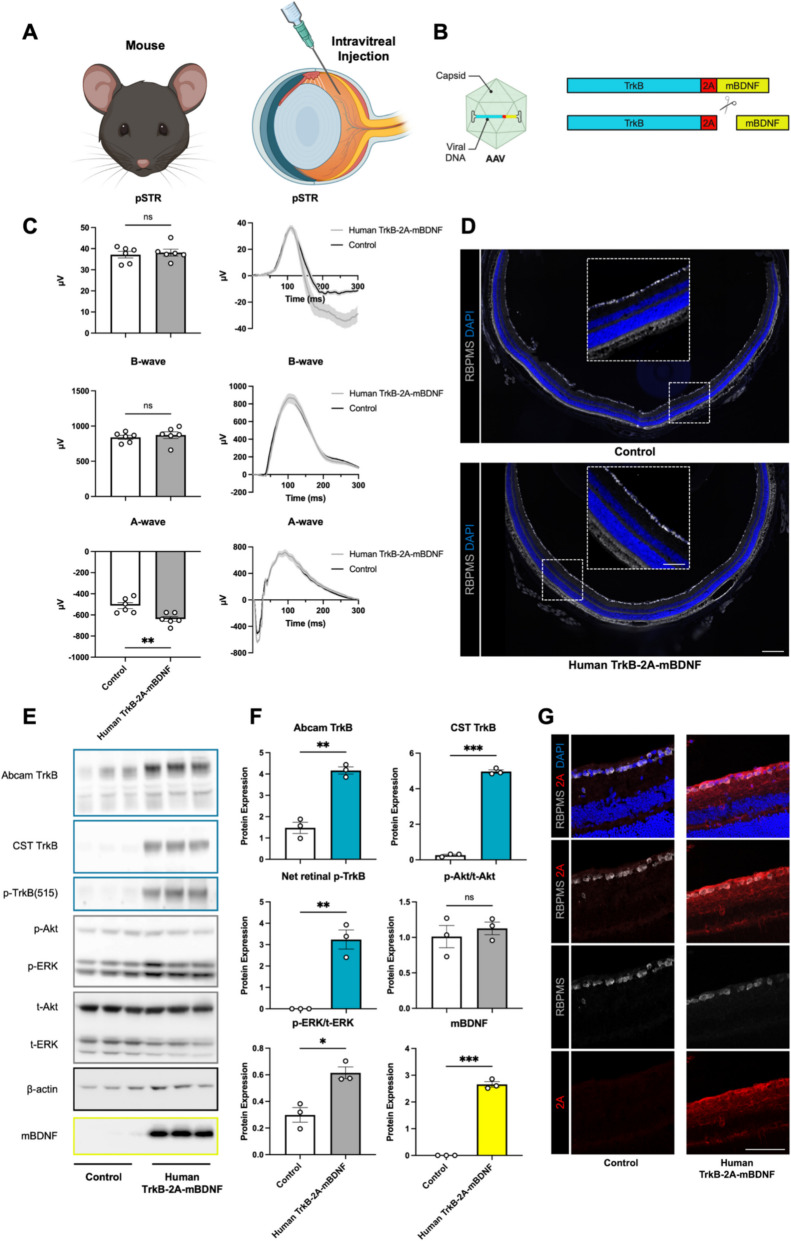
Short term safety and expression of 7.60E8 GC/eye AAV2 Human TrkB-2A-mBDNF in the murine eye 3 weeks post intravitreal delivery. A) Schematic illustration of the intravitreal injection procedure for Human TrkB-2A-mBDNF delivery into the vitreal chamber of the murine eye. B) Diagram of the AAV2 vector containing the Human TrkB-2A-mBDNF construct, which transduces retinal cells. During translation, ribosomal skipping at the 2A linker produces two distinct, fully functional proteins: TrkB and mBDNF. C) Functional ERG recordings reveal no significant impact on positive scotopic threshold responses (pSTRs), or on the B-wave. Only minor changes are observed in the A-wave, which represents photoreceptor function (n = 6). Control eyes were injected with an AAV2 Null vector. D) Retinal overviews at 5X magnification show no morphological abnormalities or damage to retinal cells post-delivery of Human TrkB-2A-mBDNF, confirming the safety of the gene therapy (n = 3). E) Western blot analysis of mouse retinal lysates 3 weeks post-injection demonstrates upregulation of the TrkB-2A-mBDNF transgene and activation of downstream TrkB signaling pathways, indicated by increased phosphorylation of TrkB, ERK, and Akt (n = 3). F) Quantification of protein expression and phosphorylation from the western blots, normalized to respective controls (n = 3). G) High-magnification (40X) images show successful transduction of retinal cells, predominantly RGCs (RBPMS-positive), with Human TrkB-2A-mBDNF with the 2A linker peptide bound to the intracellular domain of TrkB (n = 3). *p ≤ 0.05, **p ≤ 0.01, ***p ≤ 0.001 (Student’s t-test). Scale bar = 100 µm. Images A and B created in BioRender

Intravitreal administration of AAV2 Human TrkB-2A-mBDNF (Fig. 2A, B) followed by a short-term three-week expression period showed no observable adverse effects shown by functional assessments, including electroretinography, revealing no significant differences in inner retinal activity between the treated and control groups (Fig. 2C) and histological analysis revealing no signs of retinal detachment, folding, or increased immune cell infiltration near the transduced RGC layer (Fig. 2D).

Three weeks post-administration, analysis of retinal lysates demonstrated a marked increase in TrkB and BDNF protein levels relative to basal expression in the retina. These proteins were correctly translated and cleaved, producing bands at the expected molecular weights of 140 kDa for TrkB and 14 kDa for mBDNF (Fig. 2E, F; schematic in Fig. 2B). Elevated TrkB expression was confirmed using two distinct antibodies, with the CST TrkB antibody showing enhanced specificity for human TrkB compared to the endogenous rodent TrkB observed in the control group (Fig. 2E, F). Furthermore, Human TrkB-2A-mBDNF administration resulted in elevated global levels of retinal TrkB phosphorylation (p-TrkB) and activation of the downstream ERK survival signaling pathway (Fig. 2E, F), consistent with survival signaling upregulation observed in previous studies using the rodent version of the construct [17]. In contrast, in the control group, endogenous TrkB and BDNF were only detectable under overexposure conditions (Supplementary Fig. 4A), but both proteins were present in the retinal tissue.

To further confirm vector upregulated TrkB expression, an antibody targeting the residual 2A tag on the intracellular domain of TrkB was employed, as illustrated in the schematic (Fig. 2B). This antibody was absent in control eyes and successfully detected RGCs transduced with Human TrkB-2A-mBDNF (Fig. 2G). High-magnification images revealed a distinct halo-like expression pattern around the cell body, characteristic of membrane-bound TrkB (Supplementary Fig. 4B).

Specificity of the 2A tag for identifying transduced cells was further demonstrated in eyes transduced with vectors expressing eGFP-2A-mBDNF, which showed strong co-localization between eGFP and 2A (Supplementary Fig. 4C). Additional TrkB-2A-mBDNF variants displayed increased 2A expression in cells overexpressing mBDNF or TrkB, with co-detection of TrkB and 2A in transduced rat cortical neurons (Supplementary Fig. 4C, D), validating the utility of 2A tag detection for identifying TrkB-2A-mBDNF-transduced cells.

Anti-BDNF antibodies detected BDNF expression in both the nerve fiber layer and inner plexiform layer (IPL), suggesting either endogenous BDNF or potential cross-reactivity with other neurotrophins. However, retinas transduced with eGFP-2A-mBDNF provided clearer evidence of higher BDNF expression in transduced RGCs (Supplementary Fig. 4C). The secreted nature of BDNF complicates precise detection due to the difficulty in distinguishing between native and transgene-induced expression levels.

## Dose–response analysis of AAV2 Human TrkB-2A-mBDNF and neuroprotective efficacy in the murine optic nerve crush model

**Fig. 3:**
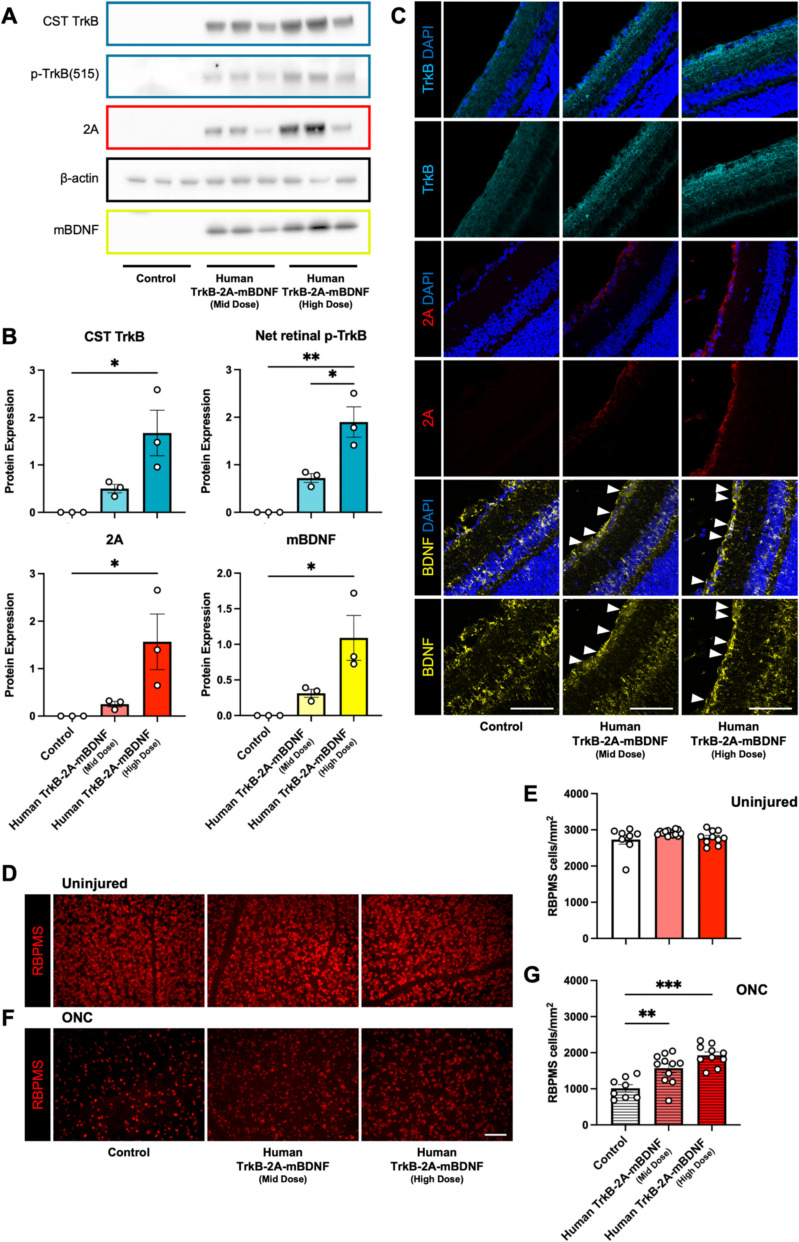
Dose reduction studies using AAV2 Human TrkB-2A-mBDNF in mice at 1.52E8 (Mid Dose) and 7.60E8 (High Dose) GC/eye and the impact on RGC neuroprotection following optic nerve crush (ONC) injury. A) Western blot analysis of mouse retinal lysates confirms upregulation of the TrkB-2A-mBDNF transgene and phosphorylation of TrkB at both the mid and high vector doses, 4 weeks post-transduction in uninjured eyes (n = 3). B) Quantification of western blot data, with protein expression levels normalized to β-actin, shows dose-dependent increases in transgene expression and overall retinal levels of p-TrkB (n = 3). C) Representative 40X images of the RGC layer show increased expression of TrkB, 2A peptide, and mBDNF at both vector doses in uninjured eyes. White arrows indicate intracellular mBDNF accumulation within RGCs prior to secretion, highlighting increased transgene activity (n = 3). D) Immunolabelling of RGCs using RBPMS markers in retinal wholemounts 4 weeks post-transduction in uninjured eyes, showing preserved RGC morphology at both doses. E) Quantification of RBPMS-positive cells across the three treatment groups (Control, Mid Dose, High Dose) in the absence of injury demonstrates no change to RGC numbers (n = 8–13). F) RBPMS immunolabelling of retinal wholemounts 3 weeks post-transduction and 1 week after ONC, illustrating RGC loss following injury. G) Quantification of RBPMS-positive cells across the three treatment groups post-ONC reveals dose-dependent neuroprotection, with significant RGC preservation in the mid and high dose groups compared to controls (n = 8–11). Statistical comparisons were also made to contralateral eyes. *p ≤ 0.05, **p ≤ 0.01, ***p ≤ 0.001 (Dunnett’s multiple comparisons test). Scale bar = 100 µm

To determine the minimum effective dose (MED) of AAV2 Human TrkB-2A-mBDNF, the vector dose was reduced five-fold in murine studies, resulting in a modest increase in protein expression compared to the endogenous levels observed in the control group (Fig. 3A, B). The high-dose group showed a significant increase in TrkB, mBDNF, and p-TrkB protein levels in comparison to the control group. However, due to the limited sample size, only phosphorylated TrkB (p-TrkB) levels displayed a statistically significant difference between the mid- and high-dose groups (p = 0.0244) (Fig. 3A, B). As expected, p-TrkB expression was undetectable in the control group, which lacked TrkB stimulation and thus reflected basal signaling levels. Western blot analysis of the 2A linker peptide revealed an expression pattern consistent with TrkB and BDNF across the different titres (Fig. 3A, B), further supporting its reliability as a marker for transduction. The 2A peptide was detected at the same molecular weight as TrkB, consistent with its attachment to the C-terminal domain of TrkB and was absent in control animals (Fig. 3A).

Immunohistochemistry confirmed elevated expression of TrkB and BDNF in the RGC layer and IPL at both dosage levels, compared to controls (Fig. 3C). The 2A marker was prominently observed in transduced cells and absent in the control group, reinforcing successful transduction with Human TrkB-2A-mBDNF (Fig. 3C).

The neuroprotective efficacy of Human TrkB-2A-mBDNF was assessed using the optic nerve crush (ONC) model. RGC counts in uninjured eyes from mice injected with mid- and high-titre Human TrkB-2A-mBDNF were comparable, indicating no toxicity from the humanized construct, similar to results observed with the Null vector injection (Fig. 3D, E, Supplementary Fig. 5A). Seven days post-ONC, RGC survival decreased significantly in all groups (Fig. 3F, G), with the control injection group showing the greatest reduction (from 2730 ± 128 RBPMS cells/mm^2^ to 1016 ± 101 RBPMS cells/mm^2^). Both mid- and high-dose Human TrkB-2A-mBDNF treatments significantly improved RGC survival compared to controls, exhibiting a dose-dependent neuroprotective effect (Fig. 3G, Supplementary Fig. 5A, B). Specifically, mid-dose Human TrkB-2A-mBDNF pre-treatment increased RGC counts by 55% (to 1573 ± 123 RBPMS cells/mm^2^), while high-dose treatment enhanced survival by 90% (to 1928 ± 92 RBPMS cells/mm^2^) relative to controls (Fig. 3G) with TUJ1 labelling also showing a preservation of RGC axons across the retina (Supplementary Fig. 5B).

## Retinal transgene expression intensity profiles for mice and rats

**Fig. 4:**
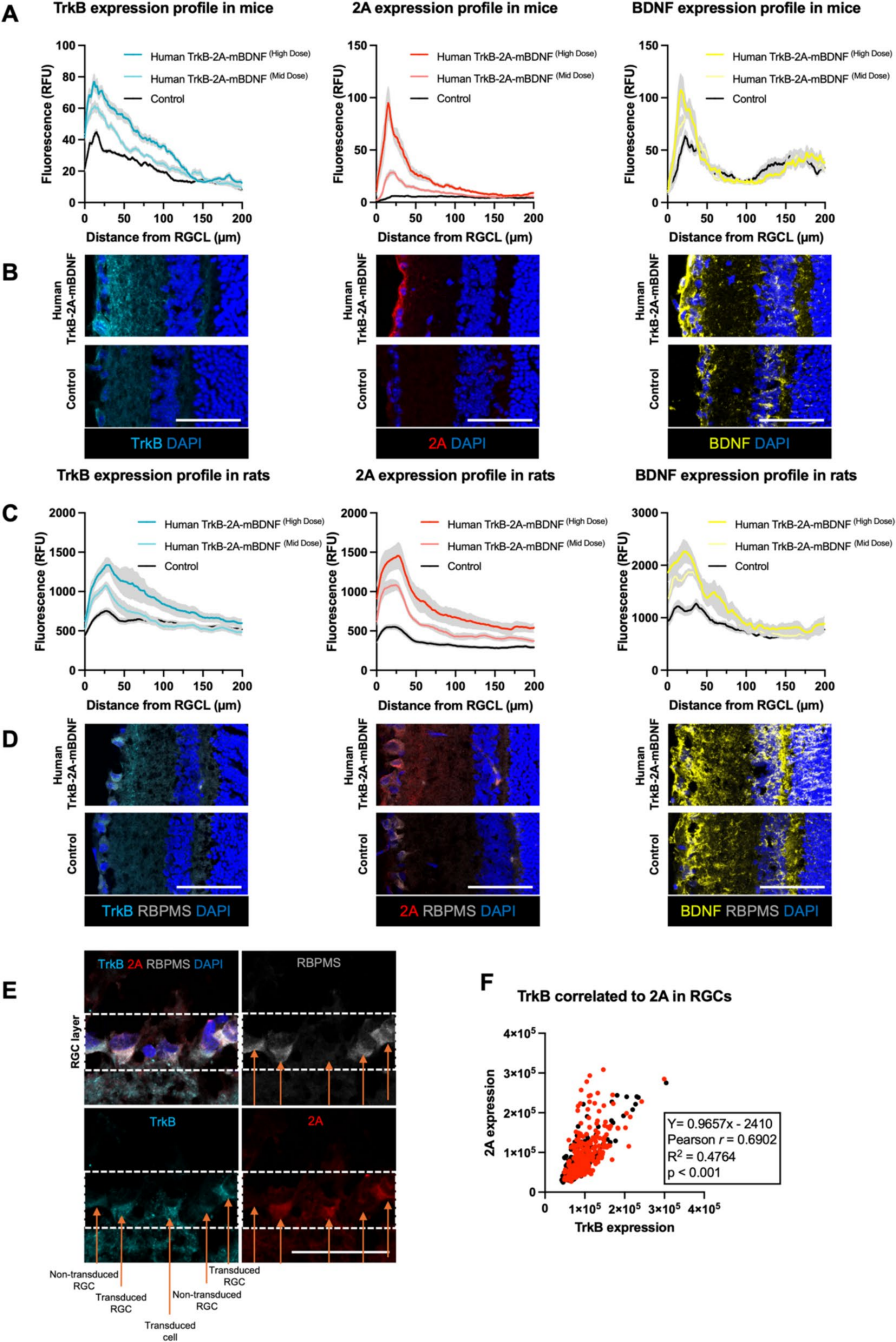
Dose studies using AAV2 Human TrkB-2A-mBDNF in mice and rats highlighting differences in protein expression profiles across the retina. A) Short term transgene expression profiles in the mouse retinal ganglion cell layer (RGCL) from the edge of the retina toward the outer retina, comparing two Human TrkB-2A-mBDNF titres to control baseline expression. Control group used an AAV2-GFP vector (7.60E8 GC/eye) (n = 3). Graphs demonstrate dose-dependent expression patterns extending across retinal layers. B) Representative images showing transgene expression across the retina in mouse eyes treated with Human TrkB-2A-mBDNF (High Dose) versus control, highlighting increased expression in treated groups. C) Transgene expression profiles in the rat RGCL extending toward the outer retina, comparing mid and high dose Human TrkB-2A-mBDNF groups to PBS-injected control eyes (n = 3). Profiles indicate a broader spread of transgene expression in rats, with distinct dose-dependent differences. D) Representative images comparing transgene expression across the retina in longer term rat eyes treated with high dose Human TrkB-2A-mBDNF versus PBS control, confirming enhanced transgene presence in treated retinas. E) High-magnification image of the rat RGC layer, 9 weeks post-transduction with high dose Human TrkB-2A-mBDNF, shows robust transduction and overexpression of both TrkB and the 2A peptide, predominantly in RGCs. F) Quantification of TrkB and 2A expression in RGCs from rat retinas transduced with Human TrkB-2A-mBDNF. Each dot represents a measured RBPMS + cell. Black dots represent cells from uninjured eyes, while red dots show measurements from eyes subjected to elevated intraocular pressure, illustrating similar expression under different conditions. (n = 6 eyes, with 445 RGCs quantified) Scale bar = 100 µm

Retinal transgene expression intensity profiles were mapped from the RGC layer to the outer retina across viral titre groups to assess vector transduction distribution. A dose-dependent increase in TrkB and BDNF expression was observed in the retinal sections of treated mice, with the highest expression levels detected in the RGC layer, as compared to native levels in control-injected eyes (Fig. 4A, B). Notably, the 2A peptide, which was absent in control-treated animals, was detected at similar distances from the inner retina in eyes receiving viral transduction (Fig. 4A, B).

**Fig. 5:**
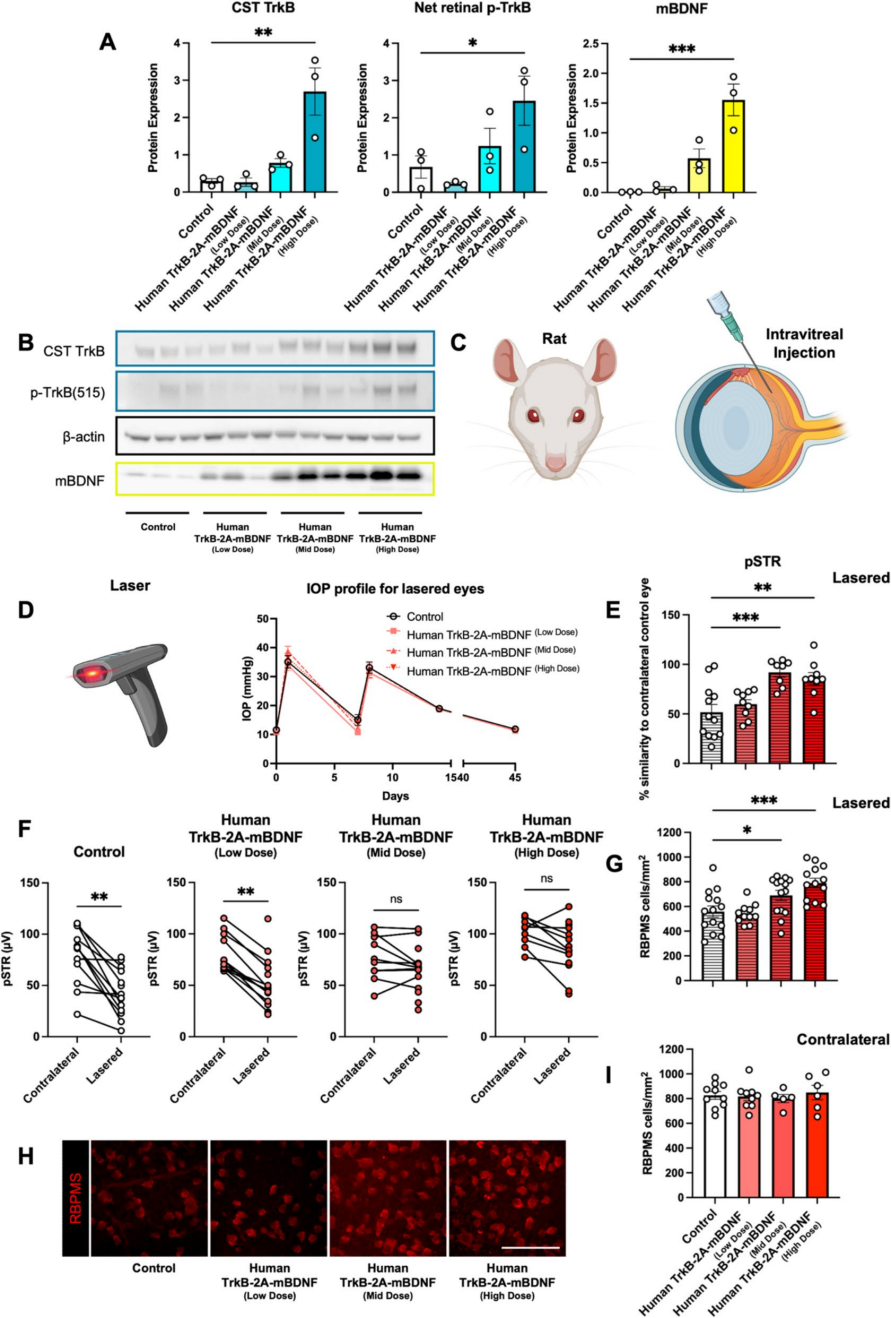
Dose ranging studies using AAV2 Human TrkB-2A-mBDNF in rats at 9.50E7 (Low Dose), 3.80E8 (Mid Dose), and 1.90E9 (High Dose) GC/eye and the impact on RGC function and preservation following elevated intraocular pressure (IOP) injury. A) Western blot analysis of rat retinal lysates 9 weeks post-transduction confirms increased protein detection of TrkB, phosphorylated TrkB (p-TrkB), and mature BDNF (mBDNF) in mid and high dose Human TrkB-2A-mBDNF groups, (n = 3). B) Western blots reveal increased band intensity for TrkB, p-TrkB, mBDNF in the top two dose groups compared to control levels (n = 3). Control animals received 5 µL intravitreal PBS injections, showing minimal endogenous protein expression. C) Schematic of the bilateral intravitreal injection procedure in rat eyes, where each animal received Human TrkB-2A-mBDNF vector delivery in both eyes. D) Three weeks post-vector administration, rats were unilaterally subjected to an ocular laser procedure, which elevated IOP to induce injury in one eye. E) pSTR measurements comparing uninjured and laser-injured eyes for each Human TrkB-2A-mBDNF dose group and controls. Graphs illustrate dose-dependent differences in RGC function between treated and control eyes post-injury (n = 8–12). F) Linked comparisons of pSTR between the uninjured contralateral and lasered eyes for each group (**p < 0.01, Student’s t-test). Each point represents individual eyes, with missing lines indicating exclusion of one eye due to poor injection scores or failed laser procedures. G) Quantification of RBPMS-positive RGCs in rat retinal wholemounts across the low, mid, and high dose Human TrkB-2A-mBDNF groups in eyes that did not undergo laser-induced injury, showing no significant difference in RGC counts compared to controls (n = 5–10). H) RBPMS immunolabelling in retinal wholemounts, 3 weeks after vector transduction and a further 6 weeks post-IOP elevation, showing RGC survival across dose groups. I) Quantification of RBPMS-positive cells across the four treatment groups 5 weeks after intraocular pressure elevation reveals a dose-dependent neuroprotective effect of Human TrkB-2A-mBDNF, with significant RGC preservation in the mid and high dose groups compared to controls (n = 11–14). Statistical comparisons were also made to contralateral eyes. *p ≤ 0.05, **p ≤ 0.01, ***p ≤ 0.001 (Dunnett’s multiple comparisons test). Scale bar = 100 µm. Images C and D created in BioRender

In rats, intravitreal administration of AAV2 Human TrkB-2A-mBDNF, scaled allometrically (see Materials and Methods, Sect. 2.5, Test Compound), produced a dose-proportional increase in transgene expression when analyzed 9 weeks post-injection, with peak expression matching for each marker at around 20-40 µm from the nerve fiber layer (Fig. 4C, D). Quantitative analysis using the area under the curve (AUC) confirmed dose-dependent elevations in transgene expression across both titre groups, with statistically significant increases in RGC layer transduction in the Human TrkB-2A-mBDNF high-dose group compared to controls (Supplementary Fig. 6A). High-magnification imaging revealed robust transduction in the rat RGC layer, with transduced cells expressing 2A (red) and displaying elevated TrkB levels (teal) (Fig. 4E). Additionally, a considerable portion of these cells co-expressed the RGC-specific marker RBPMS (grey) (Fig. 4F). Notably, in the context of elevated intraocular pressure (IOP) injury, RGCs in the injured eyes maintained the ability to upregulate TrkB and 2A, as supported by red symbols in Fig. 4F and expanded on in Fig. 5.

## Functional and structural protection over multiple doses in a rat intraocular pressure (IOP) injury model

Protein expression analysis of lysed rat retinas demonstrated a dose-dependent increase in TrkB and BDNF levels following intravitreal delivery of AAV2 Human TrkB-2A-mBDNF, consistent with the immunohistochemical findings (Fig. 5A, B). Phosphorylated TrkB (p-TrkB) levels were also significantly elevated in the high-dose Human TrkB-2A-mBDNF group (Fig. 5A, B), supporting the likely enhanced activation of TrkB signaling in the retina.

To assess the therapeutic efficacy of Human TrkB-2A-mBDNF under stress conditions, additional rats were subjected to an elevated IOP injury model three weeks post-injection, using three different Human TrkB-2A-mBDNF doses (Fig. 5C, D). IOP peaked at approximately 35 mmHg one day post-first laser (day 1) and at 33 mmHg the day after the second laser (day 8), before gradually returning to baseline levels by day 45 (Fig. 5D). This model was chosen for its reproducibility and preservation of corneal clarity, allowing for functional ERG assessment.

Six weeks post initial IOP elevation, functional analysis revealed significant differences between treated and control groups. In control-treated eyes, a substantial reduction in RGC function, as measured by the positive scotopic threshold response (pSTR), was observed between uninjured and lasered eyes (79 ± 8 µV vs 43 ± 6 µV, Fig. 5F). Rats treated bilaterally with low-dose Human TrkB-2A-mBDNF exhibited a more modest reduction in RGC function between uninjured and lasered eyes (81 ± 6 µV vs 51 ± 7 µV, Fig. 5F). In contrast, rats treated with mid- and high-dose Human TrkB-2A-mBDNF showed no significant reduction in RGC function between lasered and non-lasered eyes (Fig. 5F), indicating robust functional protection.

Quantitative analysis confirmed a 45% decrease in RGC activity in lasered control eyes (p = 0.0013, Fig. 5E, F). However, mid- and high-dose Human TrkB-2A-mBDNF-treated eyes retained nearly full inner retinal function, with minimal functional loss observed (Fig. 5E, F). ERG recordings also showed that the injury and vector treatment primarily affected the RGC layer, with negligible impact on non-RGC populations and photoreceptors in the inner and outer retina, as indicated by B-wave and A-wave measurements (Supplementary Fig. 7) which additionally confirmed the preservation of corneal clarity during laser procedures.

RGC survival closely mirrored the functional outcomes. Mid- and high-dose Human TrkB-2A-mBDNF treatment provided significant protection against RGC loss in lasered eyes (Fig. 5G, H). IOP-elevated control treated eyes exhibited approximately 32% RGC loss, while mid- and high-dose groups showed dose-dependent protection, with 25% and 40% increases in RGC counts over the lasered control group (Fig. 5G). RGC densities in the mid-dose (690 ± 0 RBPMS cells/mm^2^) and high-dose (789 ± 9 RBPMS cells/mm^2^) groups were comparable to those in uninjured eyes (820 ± 19 RBPMS cells/mm^2^, Fig. 5I).

Importantly, transgene expression persisted in RGCs of laser-injured eyes, confirming that stressed RGCs continued to produce therapeutic proteins (Supplementary Fig. 6A). This sustained expression highlights the potential of this approach for mitigating glaucomatous neurodegeneration especially given both TrkB and phosphorylated TrkB (p-TrkB) levels were reduced in the RGC layer of glaucomatous donor eyes, particularly in retinal regions corresponding to areas with clinically significant visual field loss (Supplementary Fig. 2D). These findings underscore the relevance of this strategy in restoring neurotrophic support in patients already diagnosed with glaucoma.

Finally, as a 9-week time point safety measure, minimal increases in IBA1 + inflammatory cells were observed in vector injected groups (Supplementary Fig. 6B) with slightly elevated GFAP immunoreactivity, indicative of Müller glial activation, only detected in the non-lasered retinas of the high-dose Human TrkB-2A-mBDNF group (Supplementary Fi﻿g. 6C). While MAP2 labeling of the somatodendritic compartment of neurons in the IPL remained unchanged, a trend toward increased expression in the RGC and inner nuclear layers (INL) was observed in Human TrkB-2A-mBDNF-treated eyes, suggesting enhanced neuronal survival or RGC dendrite preservation (Supplementary Fig. 6D).

## Discussion

Glaucoma remains a major unmet clinical need, as a substantial proportion of patients experience continued visual decline despite conventional IOP-lowering therapies. Although emerging neuroprotective strategies have shown early promise in clinical translation [6, 8, 9, 26], a single-administration, long-lasting treatment remains a particularly attractive approach.

Advances in gene therapies present a promising avenue for addressing this gap, particularly with the use of AAVs as gene delivery vectors. As of 2023, there were 255 ongoing or planned ocular gene therapy clinical trials for various indications, including 70 AAV trials targeting retinal diseases [27–30]. To date, no published clinical studies have explored gene therapy-based neuroprotective treatments for glaucoma [7](ClinicalTrials.gov).

Research has long demonstrated that BDNF administration can significantly enhance RGC survival, with BDNF being the most studied factor in experimental models of glaucoma (recently covered by [7, 10, 12, 31]). However, BDNF monotherapy is limited by its short half-life and poor bioavailability [32, 33]. Furthermore, the abnormal regulation of TrkB receptors in glaucomatous conditions complicates the effectiveness of BDNF alone [34, 35].

In this study, we describe the advancement of a gene therapy that combines TrkB and BDNF to offer a synergistic approach to glaucoma treatment by simultaneously addressing the limitations of BDNF monotherapy and enhancing TrkB receptor activity. The humanized TrkB-2A-mBDNF construct, was shown to be effective in expressing and remaining functional in rodent models of glaucoma. Detection methods, including measuring the protein directly or using the 2A tag attached to the intracellular domain of TrkB, may prove useful when transitioning to large animal eyes.

Importantly, this study confirms that human TrkB and mature BDNF remain compatible, with the secreted protein still able to bind and activate the downstream receptor on RGC membranes, providing protection against severe axon injury or IOP insult. The modifications to TrkB did not render the protein immunogenic in mice or rats, as evidenced by the lack of changes in visual function ERG recordings and only modest increases in glial activity, likely linked to higher dose vector-related toxicity which has been reported elsewhere [36–38].

Further studies using AAV2 TrkB-2A-mBDNF in non-human primates are anticipated to demonstrate effective protein expression levels within the range of 2E10 to 2E11 GC/eye, based on vitreal volume [39–42]. When scaled to humans, these levels align with the parameters established by previous clinical trials with similar AAV gene therapy serotypes and intravitreal delivery routes (reviewed by [29]).

## Conclusion

This study provides compelling preclinical evidence that dual gene therapy combining TrkB and BDNF expression may offer an effective neuroprotective strategy for glaucoma. Our findings build on previous research and align with recent independent work using two separate AAV vectors for TrkB and BDNF delivery, which similarly demonstrated superior efficacy when both components were co-expressed [43]. This supports the hypothesis that simultaneous activation of receptor and ligand pathways provides additive or synergistic neuroprotection, consistent with our earlier studies [16, 17].

Among the various TrkB-targeting strategies under investigation for glaucoma [13–15, 44], the bicistronic AAV vector expressing both human TrkB and mBDNF offers distinct translational advantages, including simplified dosing, controlled stoichiometry, and robust target engagement. This study represents a critical step toward clinical application, providing foundational proof-of-concept data, rodent dose–response characterization, and short-term safety assessments needed to inform larger animal studies and IND-enabling development.

Our findings of diminished TrkB expression and signaling in retinal regions vulnerable to glaucomatous damage align with previous post-mortem studies in humans [15, 35] further underscoring the therapeutic relevance of restoring TrkB activity. Taken together, these results strongly support the continued advancement of Human TrkB-2A-mBDNF as a first-in-class neuroprotective gene therapy for glaucoma.

## Study limitations

While the outcomes of this study are encouraging, several limitations should be acknowledged.

## Age of experimental animals

Glaucoma is primarily an age-related neurodegenerative disease, and the use of young adult mice and rats in this study represents a notable limitation. Age-related changes in the retina and immune response may influence both disease progression and therapeutic efficacy. Ongoing work is addressing this by evaluating the TrkB-2A-mBDNF therapy in aged animals (e.g., 2-year-old mice), with preliminary results showing similarly promising outcomes [45].

## Glaucoma models and disease relevance

The laser photocoagulation model used to elevate IOP has well-known limitations, including variability in IOP elevation and potential for procedure-induced inflammation. However, despite these caveats, it remains one of the most widely accepted and reproducible rodent models for inducible glaucoma and has been extensively used to evaluate neuroprotective strategies. To further validate the therapeutic potential of TrkB-2A-mBDNF, the construct is currently being tested across additional glaucoma models and in studies conducted by independent research groups. These efforts aim to enhance reproducibility and address potential model-specific biases.

Similarly, while the ONC model does not fully recapitulate the chronic and progressive nature of human glaucoma, it provides a reproducible and well-characterized model of acute RGC injury. Demonstrating efficacy in both ONC and laser-induced models, representing two distinct paradigms of RGC degeneration, strengthens the translational relevance of the findings and helps de-risk future progression into clinical studies.

## Transgene expression context

In this study, AAV-mediated transgene expression was evaluated in uninjured eyes for mice. While this approach offers a clean baseline for assessing transduction and safety, it would have been informative to examine expression within ONC-injured eyes. However, the severity of the crush insult and rapid RGC loss often confound such analyses, making interpretation of transduction in damaged tissue less reliable.

## Functional assessment limitations

Pattern electroretinography (PERG), which offers a more direct assessment of RGC function, was not available due to equipment constraints. As a result, only pSTR recordings were performed. While pSTR has been shown to be a sensitive and reliable proxy for inner retinal function, particularly in ONC models, the lack of PERG remains a technical limitation.

## Duration of follow-up

Another limitation is the relatively short duration of the rodent studies presented here. Long-term safety and expression were not directly assessed within this study. However, it is important to note that this research forms part of a broader portfolio supporting IND-enabling activities. Previous studies with an earlier iteration of this gene therapy demonstrated sustained expression and safety up to six months [17]. To date, there is no indication of late-onset immune responses or transgene silencing. Nonetheless, the short-term findings presented here are critical prerequisites for informing dose selection and design of forthcoming good laboratory practice (GLP) toxicology studies, which will include extended follow-up using industry-standard imaging and functional assays.

## Supplementary Information

Below is the link to the electronic supplementary material.

Supplementary file1 (DOCX 8919 KB)

## Data Availability

No datasets were generated or analysed during the current study.

## Abbreviations

AAV: Adeno-associated viral
ARVO: Association for Research in Vision and Ophthalmology’s
AUC: Area Under the Curve
AWERB: Animal Welfare and Ethical Review Body
BSA: Bovine serum albumin
CST: Cell Signaling Technology
DMEM: Dulbecco’s minimum essential medium
ERG: Electroretinography
FBS: Fetal bovine serum
GC: Genome copies
GFAP: Glial fibrillary acidic protein
GLP: Good laboratory practice
IND: Investigational new drug
INL: Inner nuclear layer
IPL: Inner plexiform layer
IOP: Intraocular pressure
ITCN: Image-based Tool for Counting Nuclei
ITRs: Inverted terminal repeats
MAP2: Microtubule-associated protein 2
mBDNF: Mature brain-derived neurotrophic factor
MED: Minimum effective dose
NFL: Nerve fiber layer
NGS: Normal goat serum
OCT: Optimal cutting temperature
ONC: Optic Nerve Crush
PERG: Pattern electroretinography
pSTR: Positive scotopic threshold response
RGCs: Retinal ganglion cells
TrkB: Tropomyosin receptor kinase B
