# Supplementary material for: Dose-ranging and further therapeutic evaluation of a bicistronic humanized TrkB-BDNF gene therapy for glaucoma in rodents
Source: Mol Neurodegener Adv. 2025 Aug 18;1(1):3. doi: 10.1186/s44477-025-00003-y (PMC12361338; doi:10.1186/s44477-025-00003-y)
Supplement: Supplementary file 1 — Supplementary file1 (DOCX 8919 KB) [file 44477_2025_3_MOESM1_ESM.docx]

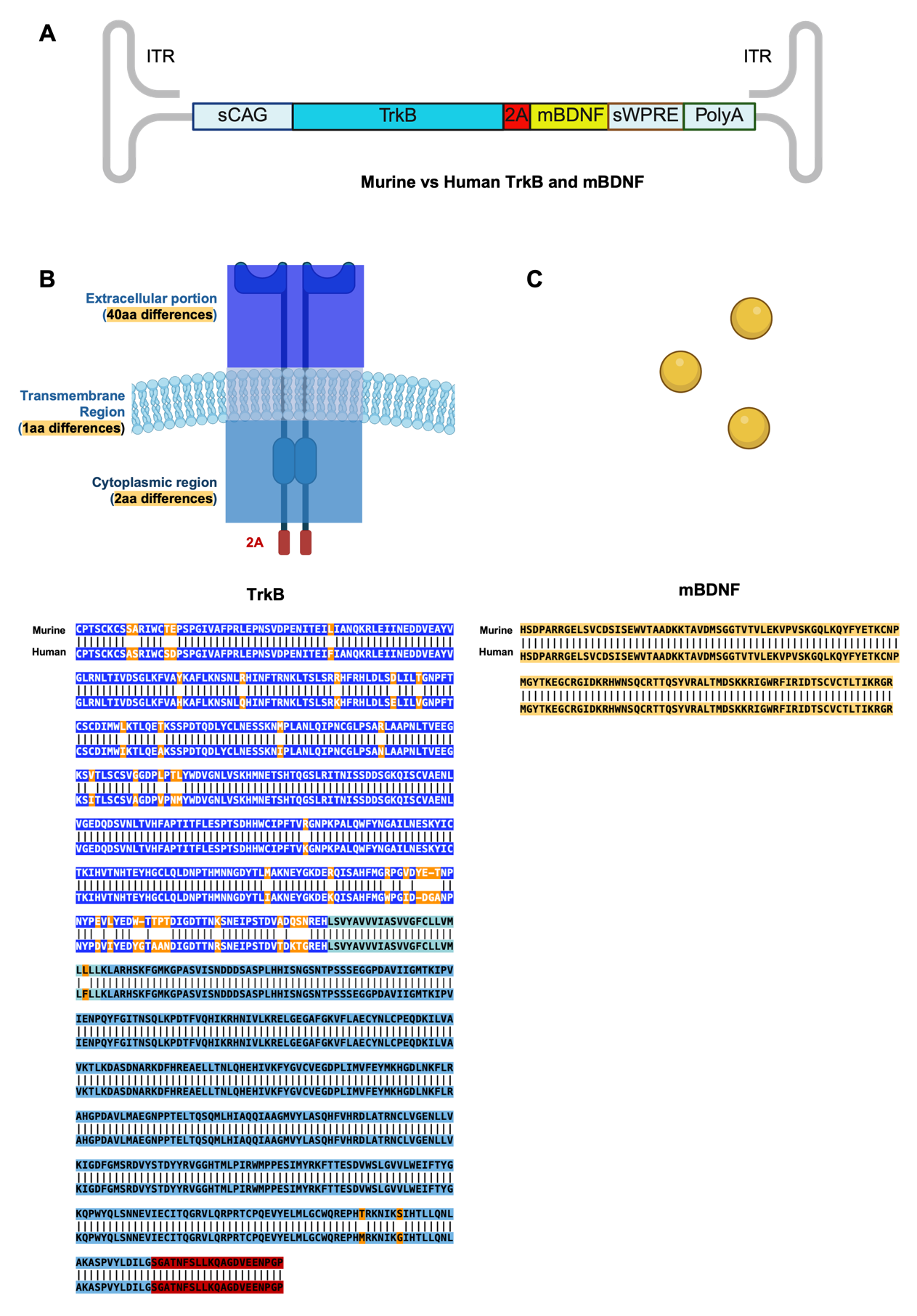


**Supplementary Figure 1:** A) AAV construct design. sCAG = shortened ubiquitous promoter, sWPRE = shortened post-transcriptional regulator. B) Schematic representation of the TrkB receptor illustrating the distinct domains and amino acid (aa) sequence differences (highlighted in orange) between murine and human proteins. Colored highlights around the TrkB receptor schematic and sequence match the extracellular, transmembrane and cytoplasmic regions. The red-highlighted sequence represents the viral 2A peptide located at the C-terminus, responsible for mediating self-cleavage during translation, resulting in the separation of the TrkB receptor from the downstream mBDNF. C) Schematic of the secreted, mature BDNF (mBDNF), with the corresponding amino acid (aa) sequence shown below, demonstrating 100% sequence homology between mice and humans. Images A to C created in BioRender.

**
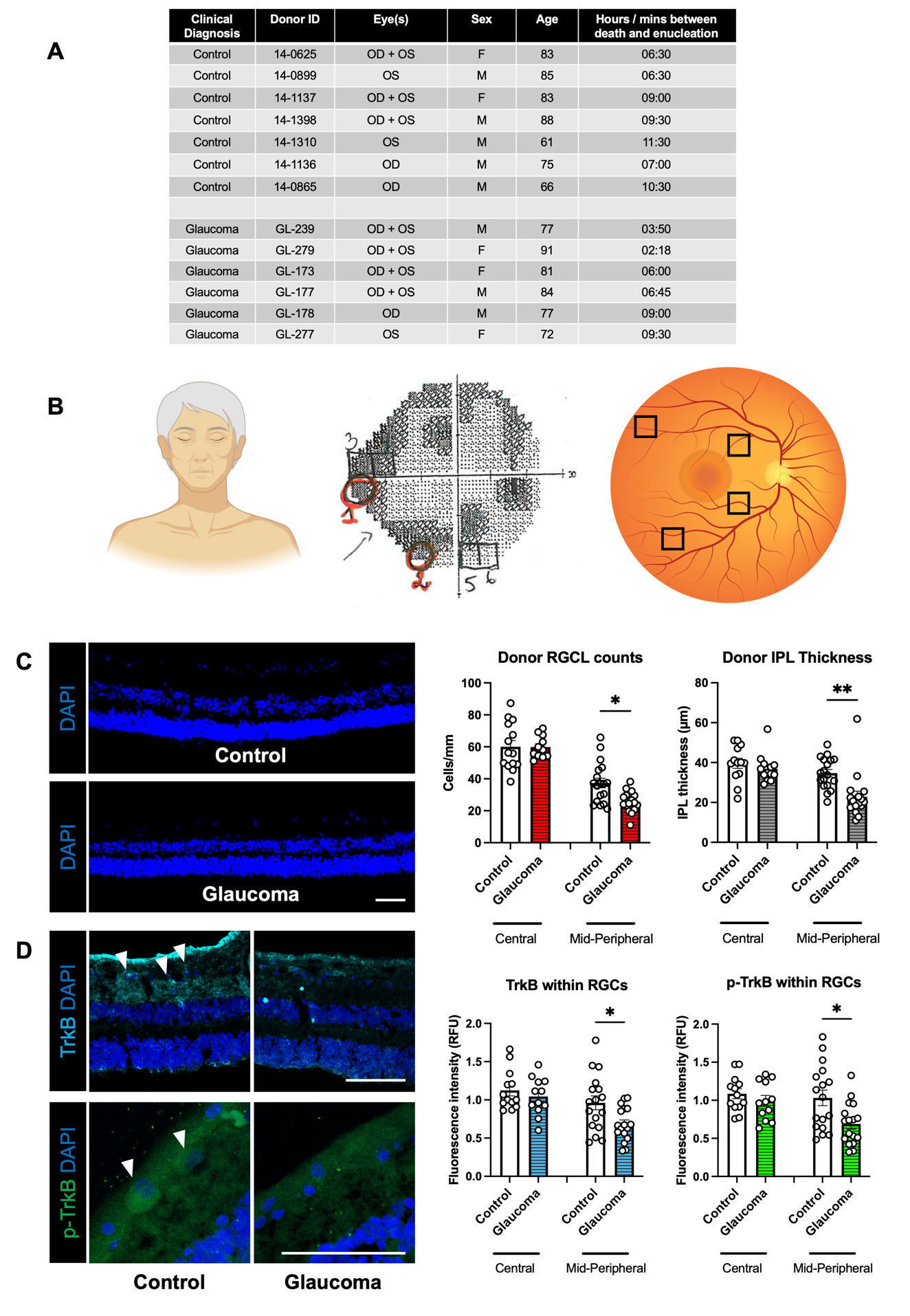
**

**Supplementary Figure 2:** Retinal sample processing for TrkB signaling from human donor patients with or without diagnosed glaucoma. A) Detailed description of human donor retinas from control and glaucoma patients. OD - oculus dexter/right eye, OS- oculus sinister/left eye. B) Representative sampling of retinal regions, including areas near the macula and regions associated with visual decline. C) Comparative analysis of retinal ganglion cell (RGC) layer counts and inner plexiform layer (IPL) thickness. While central regions of glaucoma patients showed no significant changes, mid-peripheral regions within a 30-degree arc of vision exhibited distinct morphological and structural alterations (n=11-17). D) Quantification of TrkB and phosphorylated TrkB (p-TrkB) signaling in the remaining RGCs of the mid-peripheral region. Findings revealed a significant reduction in TrkB signaling in RGCs impacted by glaucoma, whereas central RGCs retained normal TrkB expression, indicating that the differences are independent of donor variability or tissue processing artifacts (n=12–17). *p ≤ 0.05, **p ≤ 0.01 (Dunnett’s multiple comparisons test). Scale bars = 100µm. Image B created in BioRender.


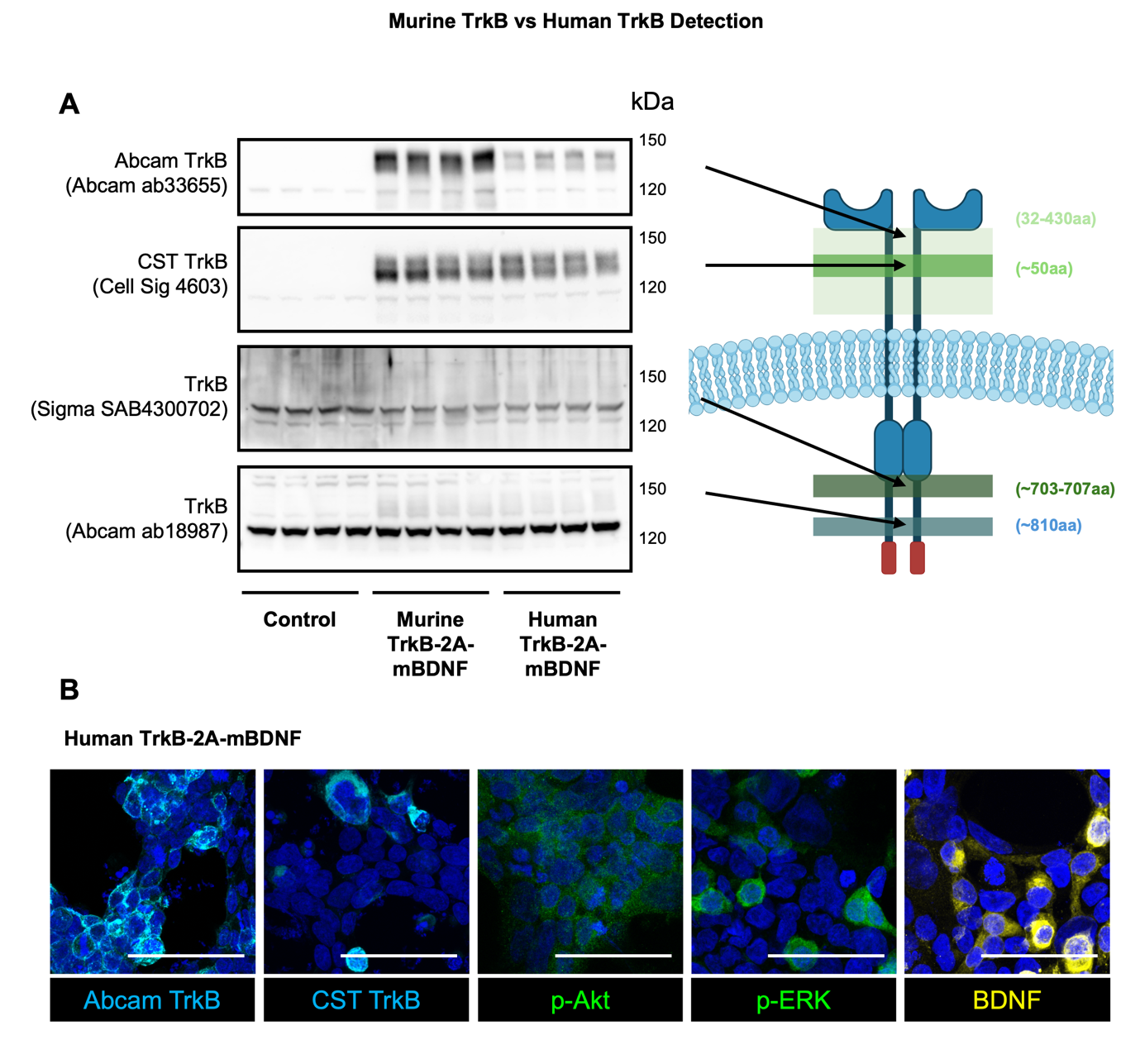


**Supplementary Figure 3:** Comparison of TrkB recognition using different commercially available antibodies targeting distinct regions of the receptor. A) Abcam TrkB (Abcam ab33655) recognized differences in the extracellular domain of the receptor between mice and human TrkB, with weaker detection for the human sequence. Antibody CST 4603 detected both murine and human TrkB proteins with comparable accuracy. Antibodies recognizing minimal amino acid (aa) sequences within the intracellular domain displayed reduced specificity, with non-specific bands detected in control HEK293T transfected cells. B) Immunolabeling of HEK293T cells with the most specific antibodies reveals the correct cellular localization of the TrkB receptor protein, activation of intracellular signaling cascades, and detection of intracellular BDNF prior to its secretion. Scale bars = 100µm. Image A created in BioRender.


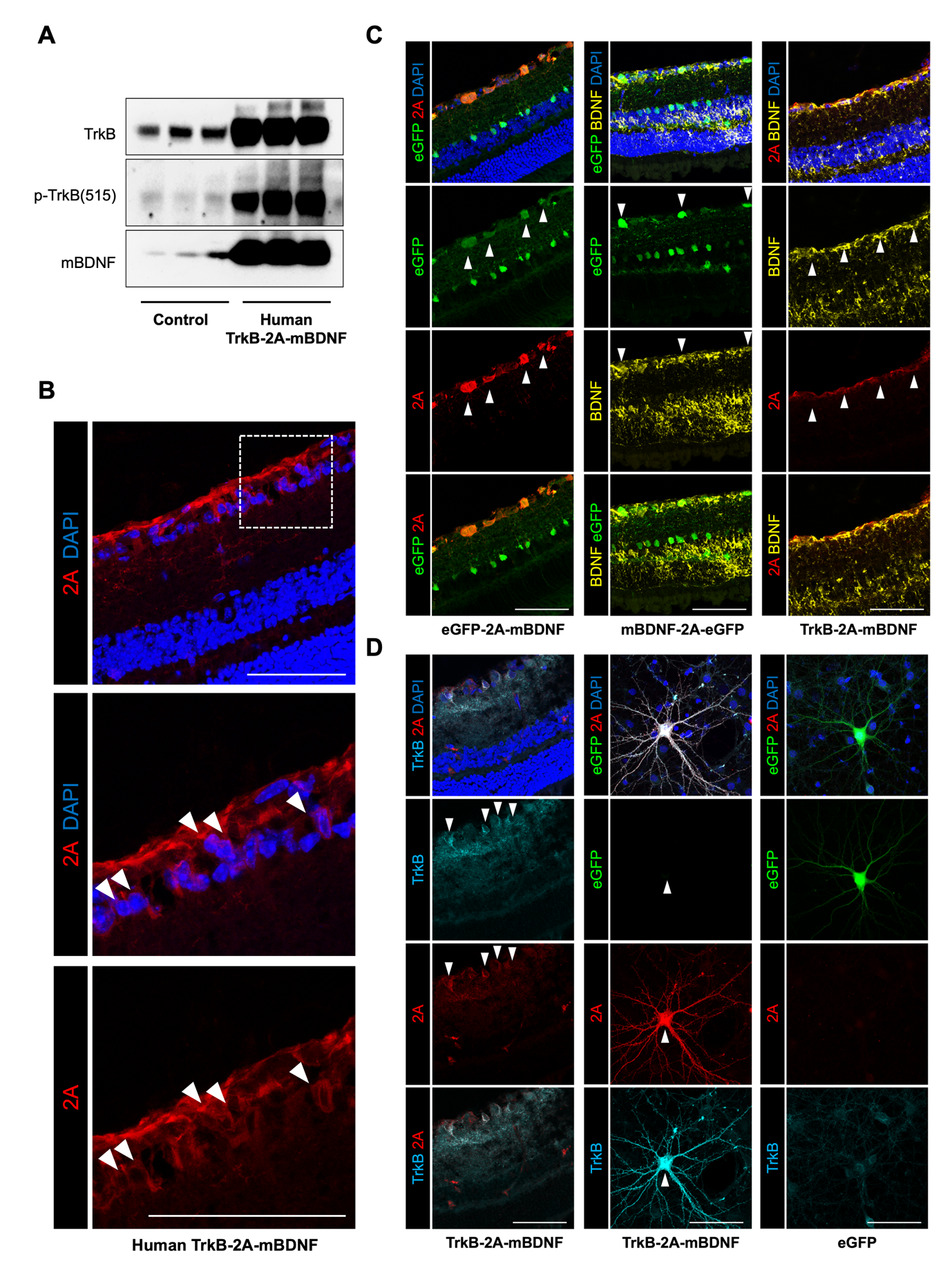


**Supplementary Figure 4:** Supporting observations of successful vector transduction. A) Oversaturated western blots illustrating basal expression levels of TrkB, p-TrkB, and mBDNF in control mouse retinas 3 weeks after intravitreal injection of AAV2 carrying either a Null or Human TrkB-2A-mBDNF vector. B) High magnification confocal images of the RGC layer in mouse retinas transduced with Human TrkB-2A-mBDNF highlight strong 2A immunolabeling, confirming vector transduction. C) Confirmation of successful transduction using an AAV2 bicistronic vector, where transduced cells (eGFP+ from the eGFP-2A-mBDNF construct) also show co-localized 2A expression. Reversing the construct orientation (mBDNF-2A-eGFP) similarly demonstrates increased BDNF expression co-localized with eGFP. The TrkB-2A-mBDNF construct shows comparable biodistribution of 2A and mBDNF expression. White arrows indicate transduced RGCs. D) The TrkB-2A-mBDNF construct shows overlapping expression profiles of TrkB and 2A in both mouse retinas and rat primary cortical neurons transduced with 7.60E8 GC/eye and 1.00E9 GC/mL, respectively. No 2A or TrkB expression is observed in AAV2-GFP transduced controls. Scale bars = 100µm.

**
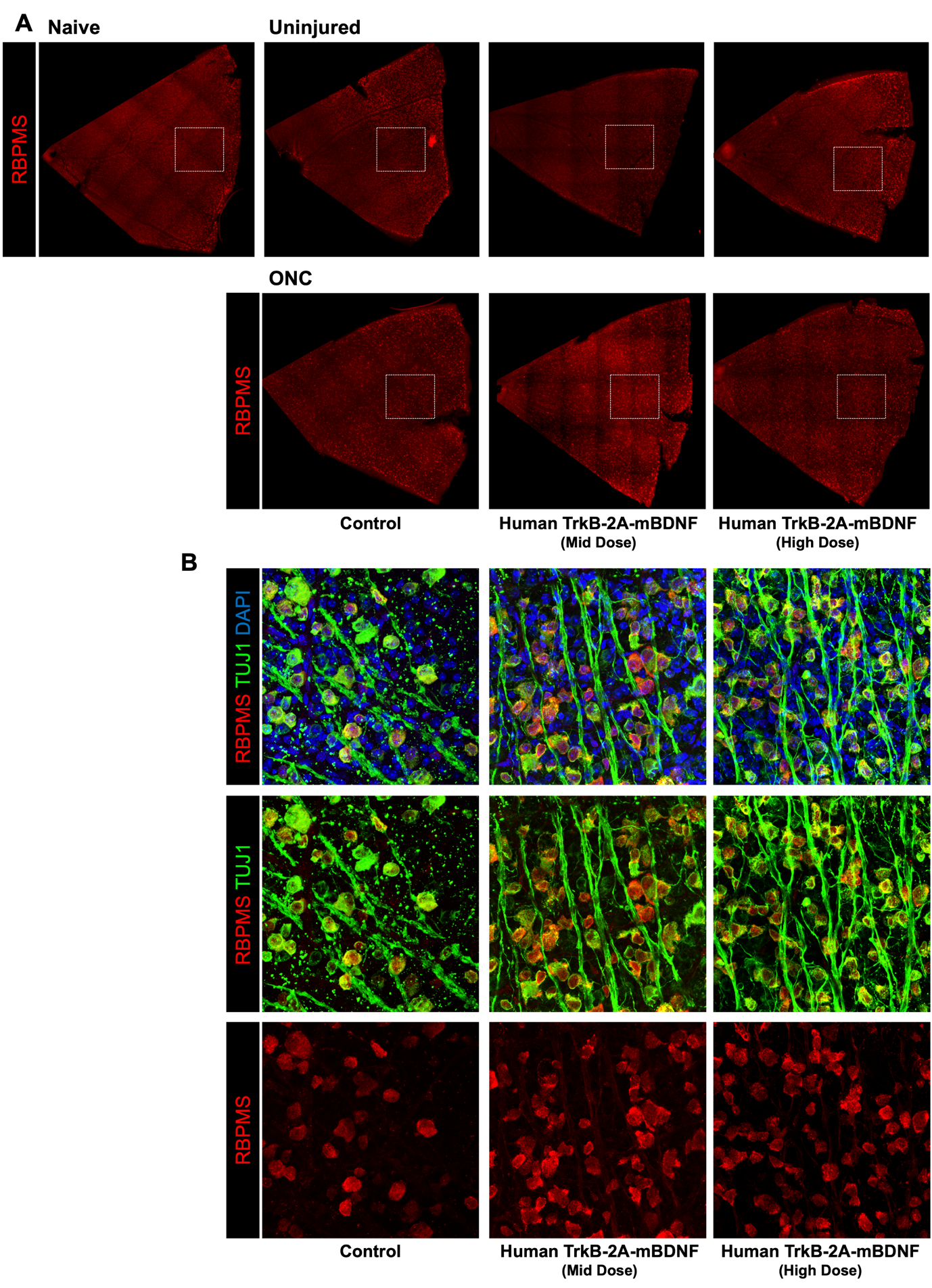
**

**Supplementary Figure 5:** Supportive observations from retinas assessed as part of the mouse ONC study. A) Representative overview images of retinal whole-mounts 7 days post-ONC for each treatment group. White boxes indicate the size of one of the eight regions analyzed for RGC quantification using RBPMS staining. B) High magnification images showing RGC markers RBPMS and TUJ1 across treatment groups following ONC injury, highlighting the differential expression and morphological changes in RGCs between groups. Scale bars = 100µm.


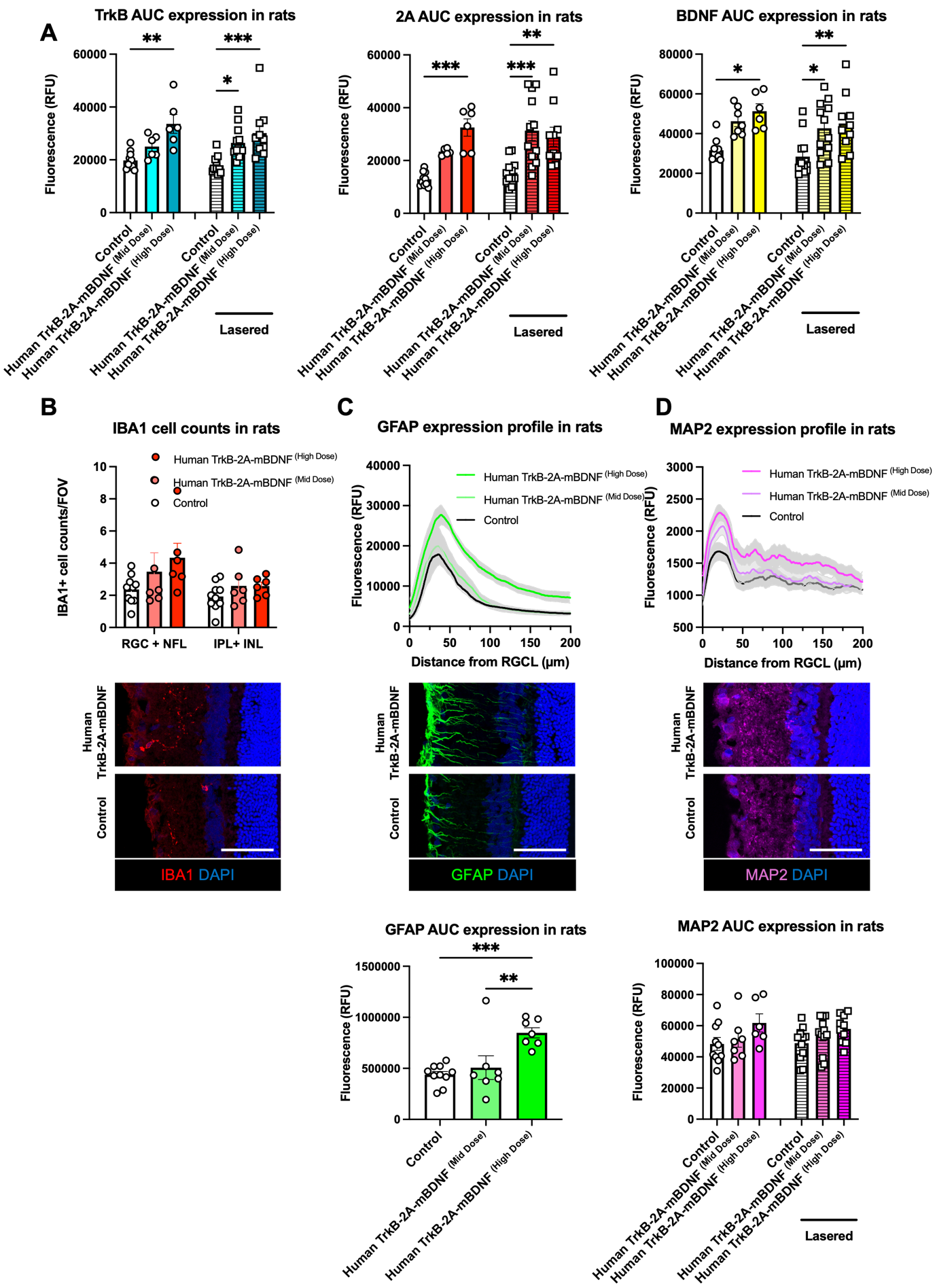


**Supplementary Figure 6:** Supportive observations for AAV2 Human TrkB-2A-mBDNF transduction in rat retinas with dose dependent expression seen in the RGC layer. A) Area under the curve (AUC) analysis of transgene expression for TrkB, 2A, and BDNF in rat retinas, comparing uninjured eyes to those subjected to laser-induced intraocular pressure (IOP) elevation, assessed 9 weeks after experiment initiation. Representative images from control and high dose Human TrkB-2A-mBDNF groups are displayed below the corresponding plots. B) IBA1-positive inflammatory cell counts were quantified in regions proximal to transduction and within deeper retinal layers, including the inner plexiform layer (IPL) and inner nuclear layer (INL). C) GFAP labeling was measured as relative fluorescence units (RFU) to assess glial activation in the inner retina. D) MAP2 labeling of somatodendritic neurons was quantified as AUC of expression within the IPL, with representative images from control and high dose Human TrkB-2A-mBDNF groups. *p≤0.05, **p≤0.01, ***p≤0.001 (Dunnett’s multiple comparisons test). Scale bars = 100µm.


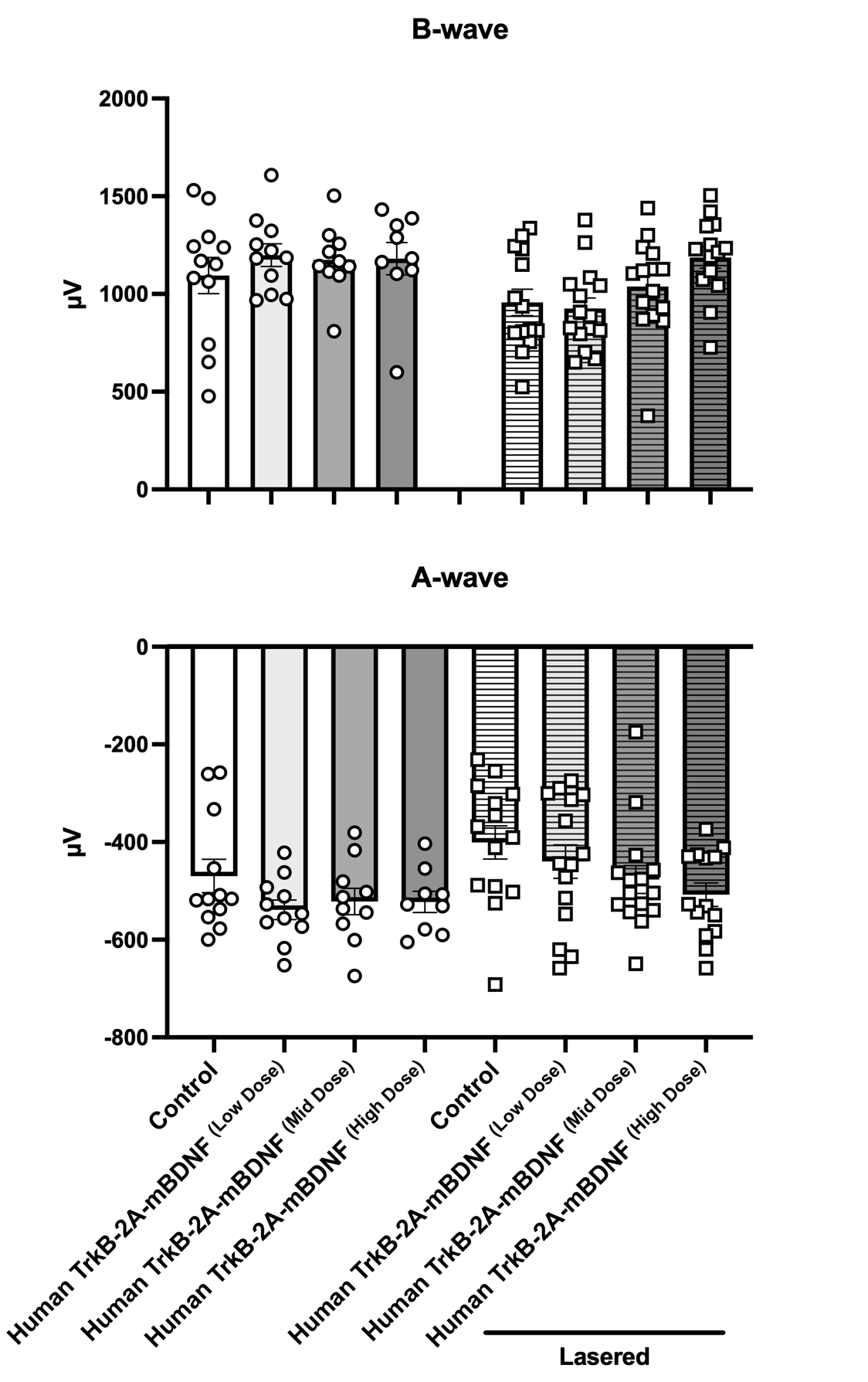


**Supplementary Figure 7:** Functional electroretinography (ERG) recordings showing B- and A-wave retinal responses following intraocular pressure (IOP) injury in rats. ERG analysis confirms selective damage to the retinal ganglion cell (RGC) population, with preserved outer retinal function.
